# Ricin and Abrin in Biosecurity: Detection Technologies and Strategic Responses

**DOI:** 10.3390/toxins17100494

**Published:** 2025-10-03

**Authors:** Wojciech Zajaczkowski, Ewelina Bojarska, Elwira Furtak, Michal Bijak, Rafal Szelenberger, Marcin Niemcewicz, Marcin Podogrocki, Maksymilian Stela, Natalia Cichon

**Affiliations:** 1 Biohazard Prevention Center, Faculty of Biology and Environmental Protection, University of Lodz, Pomorska 141-143, PL-90136 Lodz, Poland; wojciech.zajaczkowski@biol.uni.lodz.pl (W.Z.); michal.bijak@biol.uni.lodz.pl (M.B.); rafal.szelenberger@biol.uni.lodz.pl (R.S.); marcin.niemcewicz@biol.uni.lodz.pl (M.N.); marcin.podogrocki@biol.uni.lodz.pl (M.P.); maksymilian.stela@biol.uni.lodz.pl (M.S.); 2 Department of Protection Against Contamination, Military Institute of Chemistry and Radiometry, Antoniego Chrusciela “Montera” 105, PL-00910 Warsaw, Poland; e.bojarska@wichir.waw.pl (E.B.); e.furtak@wichir.waw.pl (E.F.)

**Keywords:** ricin, abrin, plant-derived toxins, ribosome-inactivating proteins, bioterrorism, field-deployable biosensors

## Abstract

Plant-derived toxins such as ricin and abrin represent some of the most potent biological agents known, posing significant threats to public health and security due to their high toxicity, relative ease of extraction, and widespread availability. These ribosome-inactivating proteins (RIPs) have been implicated in politically and criminally motivated events, underscoring their critical importance in the context of biodefense. Public safety agencies, including law enforcement, customs, and emergency response units, require rapid, sensitive, and portable detection methods to effectively counteract these threats. However, many existing screening technologies lack the capability to detect biotoxins unless specifically designed for this purpose, revealing a critical gap in current biodefense preparedness. Consequently, there is an urgent need for robust, field-deployable detection platforms that operate reliably under real-world conditions. End-users in the security and public health sectors demand analytical tools that combine high specificity and sensitivity with operational ease and adaptability. This review provides a comprehensive overview of the biochemical characteristics of ricin and abrin, their documented misuse, and the challenges associated with their detection. Furthermore, it critically assesses key detection platforms—including immunoassays, mass spectrometry, biosensors, and lateral flow assays—focusing on their applicability in operational environments. Advancing detection capabilities within frontline services is imperative for effective prevention, timely intervention, and the strengthening of biosecurity measures.

## 1. Introduction

Biotoxins are naturally occurring toxic substances produced by living organisms including plants, bacteria, fungi, and animals. These substances, often developed as a form of defense or predation, have profound biochemical effects on living organisms and, in certain cases, have been exploited by humans for both medicinal and malicious purposes. In addition to their potentially harmful effects, ribosome-inactivating plant proteins are being investigated for use in therapeutic immunotoxins and as molecular tools in scientific research. This provides an important biomedical context for this class of toxin. In addition to the risk of misuse, plant ribosome-inactivating proteins are being investigated as components of therapeutic immunotoxins and molecular tools; recent work has also reviewed vaccines and antibodies as countermeasures against ricin [1,2,3,4].

Among the vast diversity of natural toxins, plant-derived biotoxins represent a particularly important class due to their accessibility, stability, and often extreme potency [5].

Plant biotoxins exert diverse mechanisms of action depending on their molecular targets. For example, cardiac glycosides such as oleandrin, derived from *Nerium oleander* L., disrupt ion homeostasis in cardiomyocytes by inhibiting the Na⁺/K⁺-ATPase pump [6]. Alkaloids like strychnine, isolated from the strychnine tree (*Strychnos nux-vomica* L.), antagonize glycine receptors in the spinal cord, inducing convulsions and death via respiratory arrest [7]. Other potent examples include atropine (from *Atropa belladonna* L.), colchicine (from *Colchicum autumnale* L.), and coniine (from *Conium maculatum* L.) [8]. Among the most lethal and extensively studied plant toxins are ricin and abrin, produced by *Ricinus communis* L. (castor bean plant) and *Abrus precatorius* L. (rosary pea), respectively. These toxins are classified as ribosome-inactivating proteins (RIPs), which are divided into type I and type II. Type I RIPs consist of a single enzymatic chain and generally exhibit lower cellular toxicity, whereas type II RIPs, such as ricin and abrin, have a dual-chain structure comprising an enzymatic A chain and a lectin B chain, enabling cell binding and internalization, and consequently display higher cytotoxicity. Both types inhibit protein synthesis in eukaryotic cells through depurination of a specific adenine residue in the 28S ribosomal RNA [9,10,11,12,13].

Due to their exceptional toxicity and relative ease of extraction, ricin and abrin have attracted significant attention not only from the scientific and medical communities but also from defense and law enforcement sectors. Ricin is listed as Schedule 1 of the Chemical Weapons Convention (CWC), as regulated by the Organisation for the Prohibition of Chemical Weapons (OPCW), highlighting its classification as a substance with no or limited legitimate uses and a high potential for misuse in chemical warfare [14]. Their lethality of these toxins is alarming: doses as low as 1–10 µg/kg of ricin or abrin can be fatal to an adult if administered via injection or inhalation [15,16].

In recent decades, multiple instances have demonstrated the real-world use or attempted use of plant-derived toxins in terrorist attacks, political assassinations, or criminal activities. One of the most well-documented cases involving ricin occurred in 1978, when Bulgarian dissident Georgi Markov was assassinated in London by injection with a ricin-containing pellet delivered via a modified umbrella, an event that drew global attention to the covert use of biotoxins [17]. More recently, in 2003 and again in 2013, ricin-laced letters were sent to U.S. political figures including President Barack Obama and Senator Roger Wicker, though these attempts were thwarted before causing harm [17,18].

In 2018, German authorities foiled a terrorist plot in Cologne involving a Tunisian national who had successfully produced ricin with the intention of carrying out a mass-casualty attack, marking the first known case of ricin weaponization on German soil [19,20]. Similarly, in 2020, Luxembourgish intelligence services disrupted a planned terrorist attack targeting the Eurovision Song Contest in Rotterdam. The suspect had prepared a manifesto describing the use of multiple agents, including ricin, cyanide, and chlorine gas, intended for dissemination via ventilation systems and explosives. The plot was prevented before execution [21].

Although less frequently encountered, abrin has also raised security concerns. Seized materials from terrorist training camps have included abrin seeds, indicating deliberate efforts to weaponize the toxin [17]. In 2018, a man in Texas was arrested and convicted for attempting to produce abrin at home with the intent to distribute it as a weapon [22]. These incidents underscore the urgent need for sensitive and specific detection technologies capable of identifying plant-derived toxins in both environmental and clinical samples.

Notably, in 2019, Indonesian authorities thwarted a terrorist plot by the Islamic State-affiliated group Jamaah Ansharud Daulah (JAD), which planned to use abrin in a suicide bombing. Police seized over 300 g of rosary pea seeds, and toxicological analyses confirmed their potential to cause mass casualties even in microgram quantities [23]. These cases underscore the growing appeal of plant-derived biotoxins among extremist groups and the critical need for rapid, sensitive, and specific detection technologies to identify these agents in both environmental and clinical contexts.

Several factors contribute to the attractiveness of plant-derived toxins, such as ricin and abrin, for malicious use. These factors are summarized in Table 1.

From a biochemical perspective, ricin and abrin are distinguished by their modular structure, comprising a catalytic A-chain coupled with a lectin B-chain that facilitates entry into the cell. Upon internalization, these toxins inhibit protein synthesis by enzymatically depurinating a specific adenine residue within the sarcin–ricin loop (SRL) of the 28S ribosomal RNA, thereby halting translation and inducing cell death [10,25,26,27,28]. This SRL is highly conserved across eukaryotic species, which contributes to the universal toxicity of these agents.

Unlike bacterial toxins such as botulinum neurotoxin or anthrax lethal factor, plant ribosome-inactivating proteins (RIPs) do not possess dedicated delivery systems. Nevertheless, the B-chain of ricin and abrin mediates efficient receptor-dependent endocytosis, allowing these toxins to enter cells despite the absence of complex transport mechanisms. This property has important implications for detection and countermeasure strategies, as it highlights the need for systems that can identify not only the molecular presence of the toxin but also its active enzymatic function [29]. Given the dual-use potential of these biotoxins, the development of rapid, sensitive, and field-deployable detection methods has become a critical priority. Approaches include immunoassays, nucleic acid-based detection, and advanced mass spectrometry platforms. Furthermore, due to their catalytic activity and the ability of minute quantities to exert profound biological effects, methods that confirm enzymatic function are especially important for validating biological relevance in suspected samples.

This review provides a comparative overview of ricin and abrin as representative plant-derived biotoxins. While their structural and mechanistic similarities form the foundation of this discussion, we also address their real-world implications, detection challenges, and avenues for technological innovation. A comprehensive understanding of these toxins is essential not only for public health preparedness but also for global security and forensic applications.

## 2. Source, Distribution, and Epidemiology

**Figure 1:**
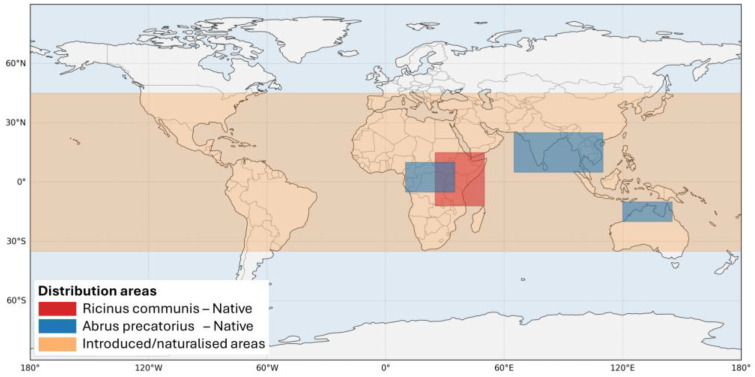
Map of the natural distribution of *R. communis* and *A. precatorius* and naturalised areas for both species.

*R. communis* and *A. precatorius* are native to tropical and subtropical regions but are now widely distributed globally due to their use in traditional medicine, ornamentation, and industrial applications (Figure 1) [30]. *R. communis* has been cultivated commercially for over 4000 years, with castor oil used in ancient Egypt as lamp fuel and in medicinal and cosmetic preparations. Today, castor oil and its derivatives are produced on a large scale for use in soaps, lubricants, hydraulic and brake fluids, paints, dyes, coatings, inks, cold-resistant plastics, waxes, polishes, nylon, and perfumes [31,32]. *A. precatorius,* commonly known as rosary pea or jequirity bean, is distinguished by its striking red and black seeds, frequently used in jewelry and decorative crafts, increasing the risk of accidental exposure, especially among children [33]. The ricin content is typically 1–5% by weight of the residual solids remaining after the oil is removed. The estimated lethal dose for humans is between 1 and 10 µg/kg. This aesthetic appeal contributes to accidental exposure, particularly in children. The plant is highly toxic because it contains abrin, a ribosome-inactivating protein that is structurally and functionally similar to ricin, yet over 70 times more potent on a weight-for-weight basis [30]. All parts of the plant are poisonous, though the seeds are most dangerous when crushed or chewed, as the hard seed coat otherwise limits absorption. The lethal dose in humans is estimated between 0.1 and 1.0 µg/kg, and ingestion of even a few seeds can result in fatal poisoning. In India and other endemic regions, *A. precatorius* poisoning is frequently linked to suicide and self-harm, with numerous case reports describing severe gastrointestinal, renal, hepatic, and neurological complications [30].

Both ricin and abrin have been identified as potential agents of bioterrorism and are classified among the most dangerous biological toxins due to their lethality and availability. Similarly, abrin’s potential misuse has prompted concerns among forensic and public health authorities, particularly given the ease of access to *A. precatorius* seeds and the lack of effective antidotes. Despite these risks, the cultivation and sale of *R. communis* and *A. precatorius* remain lightly regulated in many regions, including online marketplaces. This regulatory gap facilitates their use for both accidental and deliberate poisoning events. Strengthening toxicovigilance systems, enhancing public awareness, and promoting international cooperation on the monitoring and control of biotoxins are essential steps toward mitigating the risks associated with these plants and their derivatives [18,34,35,36].

## 3. Molecular Architecture

Ricin and abrin are highly potent toxins belonging to the class of type II ribosome-inactivating proteins (RIPs). Each toxin consists of two distinct polypeptide chains: an enzymatically active A-chain and a cell-binding B-chain, linked by a single disulfide bond. This heterodimeric architecture is essential for their biological activity. The A-chain, approximately 32 kDa in molecular weight, functions as an rRNA N-glycosylase that specifically depurinates a conserved adenine residue at position A4324 of rat ribosomes within the SRL of the 28S ribosomal RNA in the large ribosomal subunit [10,37]. This enzymatic modification irreversibly halts protein synthesis, ultimately causing cell death. Structural analyses have demonstrated that the A-chain adopts a canonical RIP fold, composed of a mixture of α-helices and β-sheets that create a catalytic cleft tailored to accommodate the target adenine base [38]. The B-chain, approximately 34 kDa, is a galactose-specific lectin responsible for binding to terminal galactose residues present on glycoproteins and glycolipids on the surface of eukaryotic cells. This interaction mediates receptor-specific endocytosis, facilitating the internalization of the toxin. The B-chain contains two homologous domains, each forming a β-trefoil structure equipped with carbohydrate-binding sites. These structural elements enable selective recognition of cell surface glycans, which differ subtly between ricin and abrin and may contribute to their differential cytotoxicity profiles [39,40]. High-resolution crystallographic analyses have confirmed that chains in ricin and abrin exhibit highly conserved tertiary structures critical for their function, underscoring the importance of these conformations in receptor binding, cellular uptake, and ribosome inactivation [40].

## 4. Cytotoxic Mechanism

**Figure 2:**
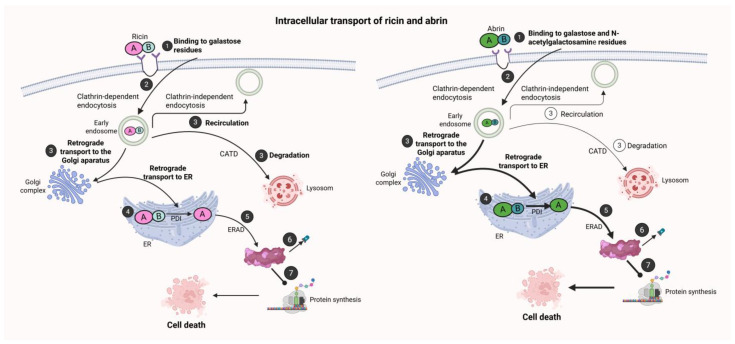
Intracellular trafficking and cytotoxic mechanism of ricin and abrin. Both toxins are heterodimeric proteins composed of a catalytically active A-chain (A) and a carbohydrate-binding B-chain (B). (1) The intoxication process begins when the B-chain binds to cell-surface glycans: ricin recognizes terminal galactose, while abrin binds both galactose and N-acetylgalactosamine. (2) Internalization occurs through clathrin-dependent and clathrin-independent endocytosis, leading to early endosome formation. (3) From endosomes, toxins may recycle to the plasma membrane, undergo lysosomal degradation, or traffic retrogradely to the Golgi apparatus and ER. (4) In the ER, protein disulfide isomerase (PDI) reduces the disulfide bond, releasing the A-chain. (5) The A-chain exploits the ER-associated degradation (ERAD) pathway to enter the cytosol. (6) In the cytosol, the A-chain depurinates 28S rRNA within the sarcin–ricin loop. (7) This blocks protein synthesis and triggers cell death. Arrow thickness reflects trafficking efficiency: ricin is more susceptible to lysosomal degradation, whereas abrin more efficiently reaches the ER, contributing to its higher cytotoxicity. Created in BioRender. Bijak, M. (2025) https://BioRender.com/7yvr5l1 (accessed on 15 September 2025) [41].

The cytotoxic effects of ricin and abrin are mediated by a complex, multistep process involving cellular entry, intracellular trafficking, enzymatic ribosome inactivation, and subsequent inhibition of protein synthesis (Figure 2). These sequential events culminate in cellular dysfunction and death.

### 4.1. Cellular Binding and Endocytosis

The B-chain’s lectin activity targets terminal galactose residues on cell surface glycoproteins and glycolipids, facilitating receptor-mediated endocytosis through both clathrin-dependent and clathrin-independent pathways [42]. The efficiency and specificity of binding are influenced by cell-type-specific glycan expression patterns, which contribute to tissue tropism and interspecies variability in toxin susceptibility.

### 4.2. Intracellular Trafficking and ER Translocation

Following internalization, the toxins undergo retrograde transport from early endosomes to the trans-Golgi network and ultimately to the endoplasmic reticulum (ER) [43]. This trafficking pathway circumvents lysosomal degradation. Within the ER lumen, the disulfide bond connecting the A- and B-chains is reduced by protein disulfide isomerase (PDI), liberating the enzymatically active A-chain [44]. Subsequently, the A-chain is retrotranslocated into the cytosol via the ER-associated degradation (ERAD) pathway. Unlike typical ERAD substrates targeted for proteasomal degradation, the A-chain evades ubiquitination and proteolysis by adopting a partially unfolded conformation conducive to cytosolic translocation [39].

### 4.3. Ribosome Inactivation and Cytotoxicity

In the cytosol, the A-chain refolds into its catalytically competent conformation and exerts RNA N-glycosidase activity by depurinating the conserved adenine residue in the SRL of 28S rRNA [10,45]. This modification prevents binding of elongation factors (eEF1 and eEF2), thereby arresting polypeptide chain elongation and effectively terminating protein synthesis. A single molecule of A-chain can inactivate thousands of ribosomes catalytically, precipitating rapid declines in protein production and triggering cellular stress responses. Cellular outcomes depend on the toxin dose and context; lower concentrations often induce apoptosis through activation of stress signaling pathways such as JNK and p38 MAPK, whereas higher concentrations result in necrosis due to ATP depletion and organelle dysfunction. Additional pathological effects include mitochondrial damage, oxidative stress, and cytoskeletal disruption observed particularly in ricin exposure [46].

Abrin shares an analogous mechanism of action with ricin but generally exhibits greater potency. While both toxins’ A-chains target the identical SRL site, differences in B-chain receptor affinity and efficiency of endosomal escape likely account for the enhanced cytotoxicity of abrin [47]. Moreover, abrin may exhibit slightly different trafficking dynamics or enhanced resistance to degradation during retrograde translocation, although this remains an area of active investigation [42].

### 4.4. Therapeutic Implications

Currently, no approved antidotes exist for abrin intoxication. However, recent studies have identified a promising neutralizing monoclonal antibody, D6F10, which binds specifically to the abrin-a isoform and inhibits its cytotoxicity [48]. Structural modeling of the abrin-a:D6F10 complex reveals multiple electrostatic interactions stabilizing the binding interface. Crucially, D6F10 sterically obstructs abrin-a’s interaction with the 28S rRNA SRL, preventing ribosome inactivation and suggesting a potential therapeutic approach.

High-resolution X-ray crystallography of abrin-A has revealed a compact tertiary structure with a catalytic cleft precisely shaped to accommodate the SRL adenine residue. Conserved active-site residues critical for N-glycosidase activity form a substrate-binding pocket analogous to that of ricin, with subtle conformational differences potentially influencing catalytic efficiency and inhibitor binding [49].

Although there is currently no licensed antidote, significant progress has been made in developing ricin countermeasures. Subunit vaccines based on the ricin A chain (e.g., RVEc and RiVax) have demonstrated acceptable safety and immunogenicity in early clinical trials, as well as providing durable protection in animal models. Correlates of protection are currently being studied in non-human primates and mice [3,50,51].

In parallel, post-exposure antitoxins, including F(ab′)_2_ preparations of equine and ovine origin, have demonstrated protective efficacy in vivo. These preparations prolong the treatment time after aerosol exposure, although marketing authorisation has not yet been obtained [4,52].

Neutralising monoclonal antibodies represent an additional pathway, and several human or humanised candidates demonstrate potent toxin neutralisation and the ability to delay treatment in preclinical systems [4]. In this context, Lequesne et al. developed antibodies that recognise both D and E isoforms of ricin and assessed their neutralising and protective potential. Two antibodies were selected—one targeting RTA (RicE5) and one targeting RTB (RicE8)—however, only RicE5 provided effective protection in an in vivo intoxication model. Administering RicE5 six hours after ricin intoxication resulted in survival rates of over 90%, while administration 24 h post-exposure achieved up to 35% survival. Additionally, mice that survived ricin intoxication through passive immunotherapy developed robust long-term immunity, a finding that may be promising for future vaccine development [53].

For abrin, antibodies under investigation (e.g., 10D8 and S008) demonstrate analogous principles at the preclinical stage. Meanwhile, small molecule-based or host-targeted approaches that disrupt intracellular transport or RTA–ribosome interactions are still in the research phase [54,55].

From a public health perspective, these measures are best viewed as complementary to early diagnosis and response. Pre-exposure vaccination may only be relevant for narrowly defined risk groups, while the benefits of antitoxins depend on rapid case identification and timely administration. Therefore, their practical impact depends on detection, orthogonal confirmation and clear reporting pathways, as described in Section 7 [3,4].

It should also be noted, however, that ribosome-inactivating plant proteins are used in legitimate biomedical applications, primarily as components of therapeutic immunotoxins and molecular tools. This issue has been documented in the literature and complements the preventive measures without changing the operational nature.

## 5. Toxicology and Stability of Abrin and Ricin

Although abrin and ricin share structural and mechanistic similarities, they differ significantly in their toxicological profiles and stability—differences that carry major implications for medical response and bio-surveillance strategies.

When it comes to relative toxicity, abrin clearly stands out as the more potent of the two. Animal studies consistently show that abrin is substantially more toxic than ricin. For example, the lethal dose 50 (LD_50_) for abrin in mice is around 10–13 nanograms per mouse via intravenous administration, compared to 55–65 nanograms for ricin [38]. Similar patterns have been observed for intraperitoneal and oral exposure, where abrin exhibits much lower LD_50_ values across a variety of species. Representative evidence includes murine i.p. dose–response studies for ricin and abrin alongside oral toxicity and clinical ingestion data that document gastrointestinal injury and systemic complications for ricin and oral-dose ranges for abrin from authoritative reviews [42,47,56,57,58,59]. This heightened toxicity is thought to result from more efficient cell binding and internalization by abrin’s B-chain, as well as its possible enhanced resistance to degradation within the cell.

Species-specific variability also plays a significant role in the toxic profiles of these biotoxins. Differences in host cell surface glycosylation—the sugar molecules that the B-chain binds to—can drastically alter susceptibility. For instance, in rat models, abrin has been found to be approximately 75 times more toxic than ricin when adjusted for body weight [38]. Such variability highlights the limitations of directly applying animal data to humans and emphasizes the importance of species-specific risk assessments.

Both toxins also demonstrate remarkable thermal and pH stability, making them particularly challenging to neutralize. Ricin retains its biological activity after exposure to mild heat and requires continuous heating at 80 °C for at least an hour in aqueous solutions to become fully inactivated. Abrin shows similar resilience, maintaining functionality despite shifts in pH and moderate heat exposure, complicating efforts to decontaminate affected areas in real-world settings [60].

Another layer of complexity is introduced by glycosylation and isoform diversity. Ricin occurs in multiple isoforms, such as ricin D and ricin E, each displaying unique glycosylation patterns that influence their solubility, immunogenicity, and behaviour inside the body. For instance, isoforms with more extensive glycosylation tend to exhibit altered cell targeting and greater stability. One study found that the toxicity of different ricin isoforms correlates directly with the degree of glycosylation, affecting both uptake and intracellular processing [61]. While less is known about abrin isoforms, it is believed they exhibit similar variability, though more comparative research is needed.

In summary, abrin’s greater potency, combined with the shared physical and chemical stability and isoform complexity of both toxins, presents significant challenges for detection, detoxification, and treatment. Understanding the isoform-specific behaviours and species-dependent toxicity responses will be key to developing more effective medical interventions and bio-surveillance tools in the future.

## 6. Detection

Accurate and timely detection of biotoxins such as ricin and abrin is critical for CBRN (Chemical, Biological, Radiological, and Nuclear) security and public safety. These highly toxic agents can be weaponized or used in targeted attacks, requiring rapid identification to initiate countermeasures and limit exposure. To meet operational demands, detection systems must combine high analytical performance with field usability.

An important milestone in biotoxin detection was the introduction in 2023 of certified reference materials (CRMs) for ricin and abrin, developed under the EU EuroBioTox project [62]. CRMs provide standardized, high-quality calibration materials that allow laboratories to accurately detect, identify, and quantify biotoxins. Prior to their availability, laboratories relied on in-house purified or commercial preparations of unknown quality, making it difficult to compare results across methods or facilities and sometimes leading to questionable data. The adoption of CRMs now enables more reliable method validation, inter-laboratory comparability, and compliance with forensic standards.

This section provides an overview of these four main analytical approaches—immunoassays, nucleic acid assays, mass spectrometry, and electrochemical biosensors—because they represent the dominant strategies reported for biotoxins such as ricin and abrin detection in CBRN response and biodefense applications.

### 6.1. Immunoassay-Based Detection of Biotoxins: Ricin and Abrin

Immunoassays remain foundational in the detection of biotoxins like ricin and abrin, owing to their specificity, sensitivity, and operational simplicity. ELISA, Electrochemiluminescence Immunoassay (ECL-IA), and Time-Resolved Fluorescence Immunoassay (TRF-IA) are the most employed formats. ELISA is particularly effective in field applications, with detection limits as low as 1–10 pg/mL. In validated studies, detection limits ranged from approximately 0.09 ng/mL in PBS to 0.28 ng/mL in whole blood, depending on the specific antibodies and assay conditions employed [63]. Comparable results have been achieved for abrin, with assay performance largely dependent on antibody quality [64].

Advanced techniques such as ECL-IA and TRF-IA provide enhanced sensitivity via signal amplification. ECL-IA can detect femtomolar toxin levels, while TRF-IA achieves high precision by separating true signals from background noise [65]. However, their instrument needs limit field deployment.

Cross-reactivity remains a challenge, notably with RCA120, a ricin-like but less toxic protein [66]. Depending on the experimental system used, the difference in toxicity between ricin and RCA120 has been reported to range from about 60- to 2000-fold [4,67]. Improvements in antibody design continue to enhance assay specificity. Overall, ELISA is the preferred tool for rapid, frontline screening, with ECL-IA and TRF-IA reserved for confirmatory or high-throughput lab settings.

Recent developments include cytotoxicity assays utilizing neutralizing monoclonal antibodies, such as MIL50 and 10D8, to detect biologically active ricin and abrin. These assays have demonstrated high sensitivity, with detection limits reaching 0.3 ng/mL for ricin and 0.03 ng/mL for abrin in complex matrices [24].

### 6.2. Mass Spectrometry for Biotoxin Detection: Ricin and Abrin

When differentiating ricin from RCA120 and other homologous proteins such as abrin, LC-ESI-MS/MS techniques are among the most effective. Both non-targeted and targeted peptide MS/MS analyses following tryptic digestion can be employed for ricin identification. In aqueous buffer, ricin exhibits high resistance to trypsin digestion unless it is first denatured and reduced. Mass spectrometry (MS), particularly LC–MS/MS, provides high analytical specificity and confirmatory capability, enabling molecular discrimination (e.g., ricin vs. RCA120) and peptide-level identification [65]. Sensitivity in complex matrices is often limited without pre-analytical enrichment; when coupled to immuno- or lectin-based affinity capture, LC–MS/MS can reach low-ng/mL levels [28,68]. By contrast, well-optimized immunoassays frequently achieve lower limits of detection in routine screening [44,45], which is why MS is best positioned as a confirmatory method following presumptive tests [63,64].

The incorporation of CRMs as calibration standards has significantly improved quantification accuracy and inter-laboratory reproducibility, addressing matrix effects and ensuring forensic reliability. Its specificity arises from detection of unique peptide fragments, minimizing false positives even in complex matrices. The method is highly reproducible, with consistent inter-laboratory results, especially when isotope-labeled standards are used [68]. Despite its strengths, MS requires sophisticated instrumentation and expert operation, making it impractical for field deployment [68,69]. Nonetheless, it remains essential for legal or forensic investigations where unambiguous identification is critical.

Portable and ambient-ionization implementations (e.g., DART-MS, paper-spray, and miniature/handheld MS) extend mass spectrometry toward near-site use. These systems enable rapid “swab-to-spectrum” screening of surfaces and powders with shorter time-to-result than laboratory LC–MS/MS, at the cost of lower resolving power and greater susceptibility to matrix effects. In layered workflows they function as triage-plus/confirmatory-adjacent tools that can accelerate escalation decisions while preserving chain-of-custody and deferring evidentiary confirmation to benchtop MS when required [70,71,72]. Method standardisation efforts (e.g., AOAC 2023.06 screening for ricin/abrin) and AI-assisted classifiers are further improving portability and interpretability in operational contexts [71,73].

Modern LC-MS/MS methods have achieved detection limits for ricin and abrin below 0.1 ng/mL, facilitating the identification of trace amounts of these toxins in diverse sample types. The specificity of MS arises from its ability to detect unique peptide fragments, minimizing false positives even in complex matrices. The incorporation of stable isotope-labeled internal standards has improved quantification accuracy and reproducibility, addressing matrix effects that previously posed challenges [74].

Zheng et al. demonstrated the successful application of antipeptide immunocapture combined with an in-sample calibration curve (ISCC) strategy for sensitive and robust LC-MS/MS quantification of clinical protein biomarkers in FFPE tumor tissues. This approach significantly improved accuracy and reproducibility in complex biological matrices, highlighting its potential for biotoxin analysis in similarly challenging sample types [75].

### 6.3. Biosensor Technologies for Ricin and Abrin Detection

Biosensors combine molecular recognition with signal transduction for rapid, sensitive detection of toxins such as ricin and abrin. These tools are increasingly applied in portable formats for use by customs, police, and emergency response teams.

Modern biosensors achieve detection limits in the picogram range. For example, aptamer-based electrochemical sensors detect ricin at 10 pg/mL, and microfluidic chips can identify abrin at 0.1 ng/mL in under 15 min [76,77].

Selectivity depends on the recognition element, with aptamers and SPR/FRET systems offering high specificity [78]. CRMs are increasingly being used in biosensor validation to benchmark sensitivity and selectivity across devices, enabling fair performance comparisons. Although environmental factors can affect reproducibility, improved materials and sensor designs now deliver consistent outputs [79].

Most biosensors are operationally simple, requiring no complex equipment. Smartphone-based and handheld devices enhance usability in the field [80]. Limitations include semi-quantitative output and challenges in non-aqueous matrices, though these are being addressed through ongoing innovation. For example Lamont et al. reported the development of a DNA aptamer specific to the ricin B chain, which was shown to efficiently extract and concentrate trace amounts of the toxin from complex liquid food matrices. The aptamer remained stable across a wide pH range, supporting its application under diverse sample conditions. When combined with surface-enhanced Raman scattering (SERS), it enabled sensitive detection of ricin not only in spiked matrices but also in intact ricin toxin, outperforming detection of the isolated B chain [81]. Another ongoing innovation is a dual-fluorescence analytical strategy that uses a nanofibre probe-mediated optofluidic dual-laser biosensor. This biosensor has been fabricated for the simultaneous detection of ricin and abrin. The nanoporous nanofibre probe (NFP) is designed to generate an evanescent wave field, enable target recognition and separation, and facilitate signal transduction. Due to size confinement, the nanopores effectively exclude toxin–aptamer complexes while retaining aptamers within the excitation zone. This assay enables the highly sensitive, parallel detection of ricin (LOD: 2.32 ng/mL) and abrin (LOD: 2.56 ng/mL) in a single operation. Eliminating complex pretreatment or additional surface modification steps conferred remarkable reusability and durability to the NFP, broadening its potential for practical applications [82].

**Figure 3:**
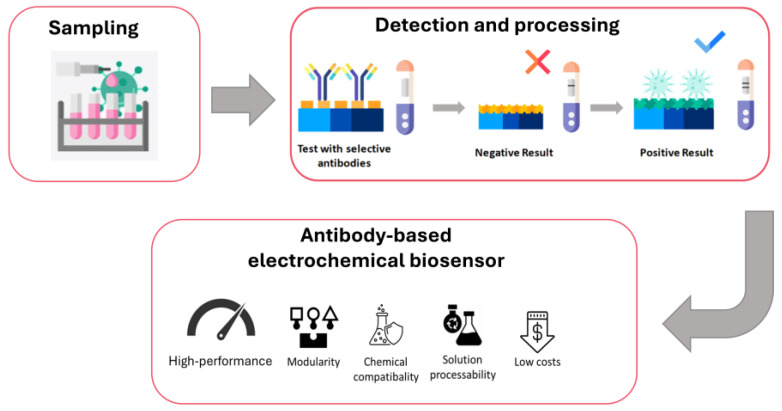
The following block diagram illustrates the process of toxin detection using an inexpensive, highly sensitive, antibody-based electrochemical biosensor. The block diagram illustrates the primary steps in the detection process, emphasising the key advantages of this detection method.

Tang et al. demonstrate a possibility to build fully operational, self-driven microfluidic chip for the simultaneous detection of ricin and abrin, offering a rapid, portable solution for field diagnostics. The device operates without the need for external pumps or power sources, relying on capillary action to drive fluid flow. It enables visual detection within 15 min, with sensitivity in the low nanogram per milliliter range (Figure 3). This simple, low-cost platform demonstrates strong potential for use in security screening, border control, and emergency response scenarios [83].

### 6.4. Lateral Flow Assays (LFAs) for Ricin and Abrin Detection

Figure 3 illustrates the primary steps of toxin detection using an inexpensive, highly sensitive, antibody-based electrochemical biosensor, highlighting the key advantages of this approach.

LFAs are compact, rapid tools ideal for field screening, typically detecting toxins at 1–10 ng/mL. While less sensitive than laboratory methods, they are portable, easy to use, and provide results in minutes [84]. As with other platforms, the introduction of CRMs supports more consistent quality control in LFA production and validation, ensuring that sensitivity claims are based on standardized material rather than variable in-house preparations. Despite these limitations, LFAs are consistent under controlled conditions. Standardized strip production and integrated controls help ensure reproducibility. Environmental factors like humidity and temperature may affect results, emphasizing the importance of proper storage. LFAs play a key role in layered bio-surveillance systems, providing immediate results that can be followed by confirmatory lab tests. Ongoing developments in digital readers and mobile integration are expanding their functionality and accuracy.

The BioThreat Alert^®^ test strips (Tetracore Inc, MD, USA) were evaluated for false positives and negatives using the following three sample types: (1) interferent performance test (PT) samples containing deionised (DI) water with calcium, magnesium, humic or fulvic acids, with or without target contaminants; (2) cross-reactivity PT samples consisting of DI water fortified with biologically or chemically similar contaminants; and (3) drinking water (DW) samples, both concentrated and un-concentrated, with or without target contaminants. For ricin, test outcomes were fully consistent across all contaminant and interferent PT samples. However, for the DW samples, variability was observed in two cases: the unspiked concentrated Florida drinking water (false positive) and the spiked New York drinking water (false negative) [85].

The abrin BioThreat Alert Test Strips were evaluated against 11 related proteins and toxins. Cross-reactivity was observed with APA-1, leading to false positives. Adding powdered milk to the buffer got rid of these false positives but also reduced how sensitive the test was to abrin [84].

Table 2 briefly summarizes the analytical and operational performance of major biotoxin detection technologies based on four key criteria: sensitivity, selectivity. Enzyme-Linked Immunosorbent Assay (ELISA) and biosensor platforms offer high sensitivity and are well-suited for field deployment due to their portability and ease of use. Electrochemiluminescence Immunoassay (ECL-IA) and Time-Resolved Fluorescence Immunoassay (TRF-IA) provide similarly high analytical performance but are restricted to laboratory settings due to instrumentation requirements. Liquid Chromatography–Tandem Mass Spectrometry (LC-MS/MS) remains the gold standard for confirmatory analysis, offering unmatched sensitivity and specificity, though unsuitable for on-site testing. Lateral Flow Assays (LFAs), while less sensitive and selective, excel in rapid triage scenarios, particularly in resource-limited or emergency contexts.

## 7. Security Needs and Response Strategies

This section translates the analytical performance of the detection platforms reviewed in Section 6 into doctrine-driven decision workflows for frontline users.

Effective protection against plant-derived biotoxins, such as ricin and abrin, hinges on frontline services’ ability to translate analytical capabilities into timely and informed decisions. The operational security layer is not just an addition to detailed biochemical information; it is an integrative backbone that links detection with prevention, consequence management and accountability within CBRN preparedness structures and international obligations [86]. In practice, end-users including border control, customs, law enforcement, forensic laboratories, public health authorities and emergency medical services need rapid, portable and reliable tools and procedures that can be used by trained non-specialists. These tools and procedures must also have clearly defined escalation paths from initial screening to confirmatory analysis and case attribution.

A layered detection concept is therefore essential. Presumptive tests (e.g., lateral flow immunoassays) can support triage within minutes and are suitable for on-site decision making. However, their known vulnerabilities, such as cross-reactivity and false positives, must be managed through predefined, orthogonal confirmation using laboratory methods, such as LC-MS/MS or functional activity assays [28]. This must be done before any legal or public health determinations are made. Mobile or near-site capabilities (e.g., peptide mapping platforms or MALDI-TOF within deployable laboratories) can shorten timelines while preserving the chain of custody and documentation standards [87]. Smartphone-assisted readers that digitise LFA outputs can reduce operator subjectivity and enable secure remote reporting to joint operations centres. These practices align with the wider CBRN doctrine of ‘screen—determine—confirm’, which prioritises fitness for purpose over analytical detection limits.

Quality assurance is the foundation of credibility. The introduction of certified reference materials (CRMs) for ricin and abrin enables harmonised calibration, inter-laboratory comparability and method validation. This replaces the previous reliance on variable in-house preparations. Building on this, the EuroBioTox consortium established an expert European network and produced multiple CRMs for priority protein toxins (including ricin and abrin), together with training and proficiency-testing resources that strengthen forensic and public-health decision-making. This governance- and QA-anchored approach ensures that operational choices remain consistent with public-health protection and non-proliferation commitments [88].

Fieldable technologies are continuing to mature, but they should only be adopted where they measurably reduce risk at operational tempos. Current frontline toolkits include lateral flow assays (LFAs), portable spectrometric techniques (e.g., ion mobility spectrometry (IMS), Raman spectroscopy and Fourier-transform infrared spectroscopy (FTIR)), targeted nucleic acid assays for contextual and selected applications of portable/ambient-ionization mass spectrometry as part of triage-plus or near-site confirmatory workflows. Selection should be based on scenario-specific performance, training load and maintainability rather than novelty alone. Promising emerging approaches include electrochemical sensors based on molecularly imprinted polymers, ambient-ionisation interfaces that expedite surface screening, and analytics that leverage machine learning to stabilise decision thresholds. These approaches are most effective when embedded within doctrines that enforce orthogonality, QA, and clear reporting semantics [70,89,90].

From a border security perspective, security requirements are translated into operational procedures at points of entry. International experience emphasises the importance of customs and border protection units in screening suspicious shipments, enforcing biosafety standards and preventing the illicit transport of plant toxins or related precursor materials. Officers are expected to follow standard operating procedures for health screening, use the appropriate personal protective equipment (PPE) during inspections and escalate any suspected toxin cases by communicating securely with national health and laboratory authorities [91]. Therefore, effective strategies require not only the deployment of technology but also interoperable cross-border information systems that ensure early warning and coordinated public health action.

Law-enforcement agencies, particularly police units serving as first responders, must be prepared to secure incident sites, protect evidence and safeguard their personnel in the event of a suspected toxin release. In this context, security needs include the ability to recognise hazards quickly, restrict access, document everything rigorously for subsequent forensic analysis and establish clear communication protocols for escalation to specialised CBRN units. Training and scenario-based exercises are essential for ensuring that police personnel can protect the public and preserve evidentiary integrity in potential bioterrorism cases. Integrating field-detection kits and mobile laboratory support can strengthen response capacity, provided such tools are used within validated workflows and supported by national forensic laboratories [92].

Emergency medical services and paramedics play a vital role in protecting lives while safeguarding themselves. They require rapid recognition of toxidromes, appropriate triage and stabilisation, decontamination procedures and the secure transfer of patients to facilities capable of providing advanced care. Protocols increasingly emphasise the necessity of personal protective equipment for responders, contamination control during transport and immediate reporting to toxicology and public health specialists. Standing orders and standardised response protocols, such as those developed for bioterrorism or chemical incidents, provide structured guidance for frontline medical responders and must be integrated into national and regional emergency medical frameworks [93].

Prevention and early warning therefore depend on more than just instruments. Intelligence-led monitoring of high-risk procurement patterns (e.g., unusual seed acquisition), routine hazard recognition training for first responders and secure, real-time data flows between customs, the police, public health laboratories and toxicology experts can close the gaps that single technologies cannot. Regular multi-agency drills with toxin release scenarios coordinated with hazmat and CBRN medical units have proven value for rehearsing triage, decontamination, sampling and information exchange at speed, and should be institutionalised with after-action learning cycles. In the European Union, recent “Cycle 12” field and tabletop exercises under the Civil Protection Knowledge Network explicitly target coordination, interoperability and robust communication/reporting protocols, offering a concrete template for doctrine-driven practice [94].

Medical management remains primarily supportive; rapid recognition, exposure documentation and early consultation with toxicologists are crucial for achieving positive outcomes and for enabling coordinated public health action. Integrating clinical pathways with forensic sampling (without compromising patient care) ensures that public health surveillance and investigative needs are met [95,96].

In summary, security needs are best met by quality-assured, interoperable systems based on a doctrine that prioritises decision-making utility over individual analytical indicators. This perspective is clearly relevant to real-world environments where ricin and abrin are present, meaning that the security response, rather than the toxins themselves, should be the organising principle of preparedness making [71].

## 8. Conclusions

Ricin and abrin are among the most potent plant-derived biotoxins, distinguished by extreme lethality, environmental stability, and the ease with which they can be extracted from widely available plants. As type II ribosome-inactivating proteins, their ability to halt protein synthesis underlies both their high cytotoxicity and their value to malicious actors. Documented use in bioterrorism and targeted criminal incidents underscores their relevance within the broader CBRN threat landscape. As outlined in Section 7, the operational stakes hinge on converting analytical capability into defensible, time-critical decisions for frontline users. Although our focus is on safety and public health response, it should be noted that the same molecular properties have also been used in legitimate biomedical research, for example, in therapeutic immunotoxins and as molecular tools. We cite these examples here to provide balance [1,2].

Significant progress has been made in detection technologies—from immunoassays and miniaturized biosensors to high-resolution mass spectrometry—yet rapid, highly sensitive, and truly field-deployable solutions remain limited. The absence of approved antidotes means that early recognition and response are critical to reduce morbidity and mortality. Since 2023, certified reference materials (CRMs) for ricin and abrin, developed under the EuroBioTox project, have provided laboratories with standardized tools for accurate detection and quantification, replacing earlier, less reliable in-house preparations [88].

Strengthening biosecurity will require systematic integration of validated detection tools into border screening, forensic workflows, and public health diagnostics, supported by interagency coordination. In practice, fitness-for-purpose follows the “screen–determine–confirm” doctrine, with orthogonal confirmation and documented QA as the backbone of decision making. Expanding toxicovigilance systems, enhancing analytical capabilities, and embedding scenario-based exercises into training programs are essential to preparedness [94].

Addressing the evolving threat posed by ricin, abrin, and other emerging biotoxins demands a sustained, multidisciplinary, and globally coordinated effort that links scientific innovation with operational readiness. Evaluating emerging approaches against predefined decision use-cases (e.g., points-of-entry triage, postal screening, scene control) will help translate innovation into interoperable, quality-assured workflows that perform at operational tempo.

## Figures and Tables

**Table 1:** Principal attributes contributing to the attractiveness of ricin and abrin for malicious use.

Factor	Description
Accessibility	Plants producing ricin and abrin are widely distributed in tropical and subtropical regions and can be cultivated with minimal expertise.
Ease of extraction	Simple mechanical or aqueous extraction methods can yield crude toxins with high biological activity [24].
Lack of detection infrastructure	Many security screening systems are not equipped to detect biotoxins unless specifically targeted.
Stability	These toxins remain stable under various environmental conditions, enhancing their persistence and effectiveness during dissemination.

**Table 2:** Comparative overview of detection methods for ricin and abrin.

Detection Method	Sensitivity	Selectivity	Ref.
ELISA	0.09–0.28 ng/mL in PBS/blood	Antibody-dependent; potential cross-reactivity (e.g., RCA120)	[42,43,44,45,46,47,48,49,50,51,52,53,54,55,56,57,58,59,60,61,62,63,64,65,66,67,68,69,70,71,72,73,74,75]
ECL-IA/TRF-IA	femtomolar–sub-ng/mL	High *	[45]
LC-MS/MS	<0.1 ng/mL after immuno/lectin capture **	Excellent (confirmatory)	[42,48,49,50]
Biosensors	10 pg/mL–0.1 ng/mL depending on platform	Recognition-element dependent	[52,53,54,55,56,57]
LFAs	1–10 ng/mL	Moderate; antibody-based	[58]

(*) antibody-based detection method. (**) with affinity enrichment. Reported LC–MS/MS limits of detection for ricin/abrin typically rely on pre-analytical immuno- or lectin-affinity capture [28,68].

## Data Availability

No new data were created or analyzed in this study.

## Abbreviations

The following abbreviations are used in this manuscript:

AI	Artificial Intelligence
APCI	Atmospheric Pressure Chemical Ionization
CBRN	Chemical, Biological, Radiological, and Nuclear
CRM	Certified Reference Material
ECL-IA	Electrochemiluminescence Immunoassay
ELISA	Enzyme-Linked Immunosorbent Assay
ER	Endoplasmic Reticulum
ERAD	Endoplasmic Reticulum-Associated Degradation
eEF	Eukaryotic Elongation Factor
FFPE	Formalin-Fixed Paraffin-Embedded
FTIR	Fourier-Transform Infrared
JAD	Jamaah Ansharud Daulah
LC-ESI-MS/MS	Liquid Chromatography–Electrospray Ionization–Tandem Mass Spectrometry
LC-MS/MS	Liquid Chromatography–Tandem Mass Spectrometry
LFA	Lateral Flow Assay
MAPK	Mitogen-Activated Protein Kinase
MALDI-TOF	Matrix-Assisted Laser Desorption Ionization-Time of Flight
MIRM	Multiple Isotopologue Reaction Monitoring
ML	Machine Learning
MIP	Molecularly Imprinted Polymer
MS	Mass Spectrometry
OPCW	Organization for the Prohibition of Chemical Weapons
PBS	Phosphate-Buffered Saline
PCR	Polymerase Chain Reaction
PDI	Protein Disulfide Isomerase
RIP	Ribosome-Inactivating Protein
RCA120	Ricinus communis Agglutinin 120
RCSB	Research Collaboratory for Structural Bioinformatics
SRL	Sarcin–Ricin Loop
SPR	Surface Plasmon Resonance
TRF-IA	Time-Resolved Fluorescence Immunoassay
